# Clinical Cases

**Published:** 1882-11

**Authors:** E. W. Berridge

**Affiliations:** London


### CLINICAL CASES.

E. W. BERRIDGE, M. D., LONDON.

*Silicea* in vertigo.—Miss L., aged between thirty and forty. I saw her at 8.40 A. M. Since 4.30 A. M. she had had vertigo, as if all things were going round; worse on lying down, especially *when lying on left side;* better by rising from lying, but returning soon after lying down again. Silica Cm (F. C.), one dose. After this dose there was no return of the vertigo, even when lying. *Silicea* is the only remedy known at present to produce vertigo *when lying on left side.* The symptoms were produced in a proving with the 2d, 3d and 4th potencies, and cured with the Cm potency. This case shows that organopathic Sharp's theory of regulating the potency of the remedy by the potency which produced the similar symptoms is untenable. Hahnemann gives us rules in his *Organon*, both for the regulation of the potency and the repetition of the dose, and these rules have never been refuted.

*Arum triphyllum* in sequelæ of typhoid.—Sept. 26th. A child, nine years old; seventeenth day of typhoid, which had been treated allopathically. *Bores violently in nostrils*, especially the left. *Bites her nails.* Purple spot on outside of left nose. Emaciation. Cannot sleep. Tongue white, with red spots, especially red round the tip. Throws the clothes off herself. Urine scanty. Voice affected. I never saw this patient, but prescribed from the report sent me by a friend, *Arum triphyllum* 20m (Fincke) every four hours. (See Lippe's *Repertory*, p. 54.)

September 28th. Better; urine more copious; sleeps much better; still bores in nostrils. No medicine.

September 30th. Sleeps well; voice returning; urine free; bowels relieved yesterday. Has not picked her nose to-day, nor bitten her nails so much; not nearly so much boring in nose.

October 3d. Sleeps well; voice stronger; no boring in nose; less biting of nails; urine free; no stool since 30th; has taken beef tea; is stronger. The purple spot on nose has discharged pus. No further detailed report, but heard that she recovered.

